# The first fatal case of *Corynebacterium ulcerans* infection in Japan

**DOI:** 10.1099/jmmcr.0.005106

**Published:** 2017-08-10

**Authors:** Ken Otsuji, Kazumasa Fukuda, Takeru Endo, Satoko Shimizu, Nobuya Harayama, Midori Ogawa, Akihiko Yamamoto, Kaoru Umeda, Toshiyuki Umata, Hiroyuki Seki, Masaaki Iwaki, Masayuki Kamochi, Mitsumasa Saito

**Affiliations:** ^1^​ Department of Microbiology, School of Medicine, University of Occupational and Environmental Health, Kitakyushu, Japan; ^2^​ Department of Critical Care Medicine, Hospital of the University of Occupational and Environmental Health, Kitakyushu, Japan; ^3^​ Department of Bacteriology II, National Institute of Infectious Diseases, Tokyo, Japan; ^4^​ Department of Microbiology, Osaka City Institute of Public Health and Environmental Sciences, Osaka, Japan; ^5^​ Radioisotope Research Center, Facility for Education and Research Support, University of Occupational and Environmental Health, Kitakyushu, Japan; ^6^​ Department of Laboratory and Transfusion Medicine, Hospital of the University of Occupational and Environmental Health, Kitakyushu, Japan

**Keywords:** *Corynebacterium ulcerans*, pseudomembrane, dyspnea, asphyxia, fatal, ECMO

## Abstract

**Introduction.** *Corynebacterium ulcerans* (*C. ulcerans*) is a zoonotic pathogen that occasionally causes diphtheria-like symptoms in humans. Cases of *C. ulcerans* infection have been increasing in recent years, and *C. ulcerans* has been recognized as an emerging pathogen.

**Case presentation.** Here we report a case of asphyxia death due to pseudomembrane caused by diphtheria toxin (DT)-producing *C. ulcerans.* This is, to our knowledge, the first fatal case of *C. ulcerans* infection in Japan. A strain of *C. ulcerans* was obtained from the patient’s pet cat and was confirmed to be identical to the patient’s isolate by sequencing of the 16S rRNA gene and the DT gene, by pulsed-field gel electrophoresis (PFGE) and by ribotyping. In the same way, it was revealed that the isolate in this case belonged to the same molecular type as the *C. ulcerans* 0102 isolated from the first case in Japan in a distant prefecture 15 years earlier, in 2001.

**Conclusion.** DT-producing *C. ulcerans* can be contracted from a companion animal and causes human death if the appropriate treatment is delayed. The finding indicates that this molecular type of virulent *C. ulcerans* is currently widespread in Japan.

## Abbreviations

CT, computed tomography; DAT, diphtheria antitoxin therapy; DT, diphtheria toxin; DTaP-IPV, diphtheria, tetanus, acellular pertussis-inactivated polio vaccine; VA ECMO, veno-arterial extracorporeal membrane oxygenation; PFGE, pulsed-field gel electrophoresis.

## Introduction

*Corynebacterium ulcerans* is a commensal in animals and was primarily known as a bacterium that causes mastitis in cattle [1]. Some strains of *C. ulcerans* produce diphtheria toxin (DT). Though human infections are rare, the strains occasionally cause tonsillitis, pharyngitis, sinusitis, pneumonia and peritonitis in humans. The frequency and severity of human infections associated with *C. ulcerans* has been increasing in the last 20 years, and recently *C. ulcerans* has been recognized as an emerging human pathogen [2]. We experienced a case of asphyxia death due to pseudomembrane caused by DT-producing *C. ulcerans* in 2016. This is, to our knowledge, the first fatal case of *C. ulcerans* infection in Japan. Here we report the clinical course of the case and the results of bacteriological analysis of the *C. ulcerans* isolated from the patient.

## Case report

**Fig. 1.**
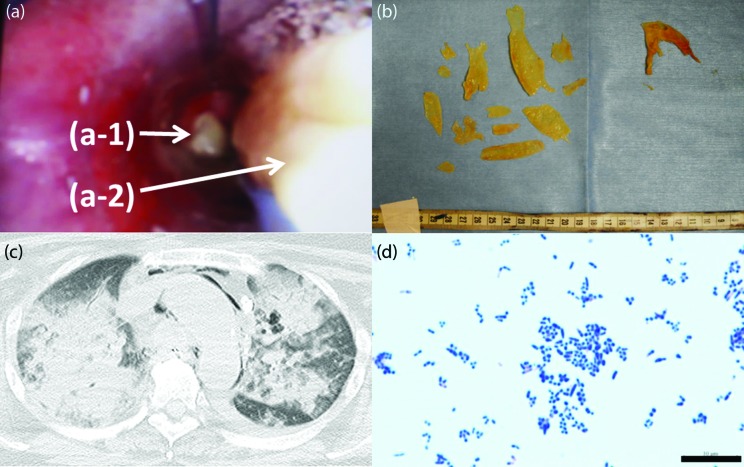
Observation of the tracheal lumen using a bronchoscope (a); Tracheal bifurcation is obstructed by pseudomembrane (a-1). Pseudomembrane was sampled with forceps (a-2). The fragments of pseudomembrane sampled by forceps (b). Thoracic computed tomography (CT) shows bilateral consolidations in the lung field, pneumomediastinum around the aortic arch and obstructed tracheal lumen (c). Gram staining (×1000) of organisms isolated from the pseudomembrane sample. Bar: 10 µm (d).

In late spring of 2016, a 66 year-old Japanese woman, who had complained of dyspnea for a week, was taken by ambulance to the hospital of the University of Occupational and Environmental Health. The patient was under medication in a municipal hospital for depression, diabetes, hypertension and hyperlipidemia. She lost consciousness in the ambulance and was in a state of cardiopulmonary arrest after her arrival at the hospital. Although cardiopulmonary resuscitation, including endotracheal intubation, was performed, her state of disabled ventilation continued, so she was admitted to the intensive care unit for treatment with percutaneous veno-arterial extracorporeal membrane oxygenation (VA ECMO). A large amount of pseudomembrane was observed in her tracheal lumen through the bronchoscope, and it obstructed her tracheal bifurcation (Fig. 1a, b). Thoracic computed tomography (CT) images showed bilateral consolidations in the lung field, pneumomediastinum around the aortic arch, and obstructed tracheal lumen (Fig. 1c). Suspension of the pseudomembrane was aerobically cultured at 37 °C on sheep blood agar. After overnight culture, white colonies appeared and a Gram stain (×1000) of the colonies showed Gram-positive bacilli (Fig. 1d). As she was suspected of having respiratory diphtheria infection, penicillin was administered and the pseudomembrane was removed as much as possible to improve her ventilatory condition. Despite these treatments, her cardiorespiration did not improve and she died on the third day after hospitalization.

The bacterium isolated from the pseudomembrane was identified as *C. ulcerans* (API code 0111326 ID99.7 %) by an API Coryne system (SYSMEX bioMérieux). DNA extraction was performed from the colonies to determine the 16S rRNA gene sequence. A partial sequence (1443 bp) of the 16S rRNA gene was similar (>99 % identities) to that of *C. ulcerans* NCTC 7910, which is a type strain of *C. ulcerans* (GenBank accession number X84256).

**Fig. 2.**
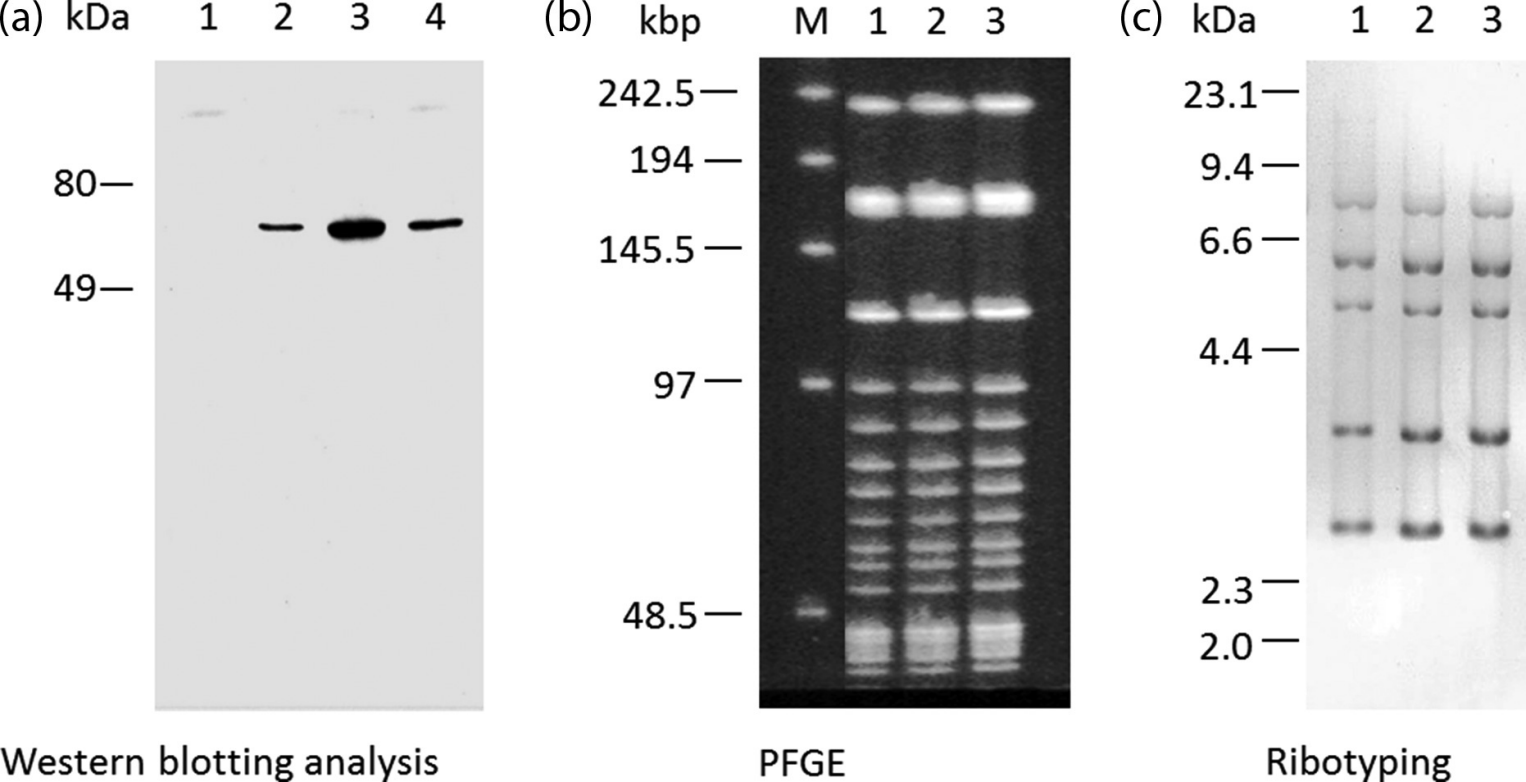
The result of Western blotting analysis using anti-DT antibody (a). Sizes are indicated on the left. Lanes: 1, supernatant of fresh culture medium; 2, purified DT (0.1 ng); 3, culture supernatant of *C. ulcerans*; 4, culture supernatant of DT-producing *Corynebacterium diphtheriae*. SfiI/PFGE result of *C. ulcerans* strain 0102 and strains isolated in this study (b). Lanes: M, lambda marker (sizes are indicated on the left); 1, strain 0102; 2, the isolate from the patient; 3, the isolate from the patient’s pet cat. Ribotyping result of *C. ulcerans* strain 0102 and strains isolated in this study (c). Marker sizes of lambda Hind III are indicated on the left. Lanes: 1, strain 0102; 2, isolate from the patient; 3, isolate from the cat.

The diphtheria toxigenicity of the isolate was evaluated by PCR of the DT gene, Western blotting analysis using anti-DT antibody, and a Vero cell cytotoxicity test. The PCR analysis [3] was positive for the DT gene (1585 bp, data not shown). The expression of the DT protein was confirmed by Western blotting analysis using a method described previously [4] with a modification (Fig. 2a). As a result of the Vero cell cytotoxicity test described previously [5, 6], the isolates were confirmed to have Vero cell cytotoxicity, which was inhibited by the anti-DT antibody (Table 1). From these results, it was concluded that the isolates were DT-producing *C. ulcerans*.

**Table 1.** Characteristics of the isolates of *C. ulcerans* and *C. diphtheriae* strain PW8

Species	Source	API-Coryne code (probability %)	16S rRNA gene (1443 bp, percentage identity)	DT PCR (1585 bp)	Cytotoxicity titer (Vero CD_50_/25 µl)
*C. ulcerans* (patient)	Pseudomembrane	0111326 (99.7 %)	*C.* *ulcerans* (99.5 %)	positive	1×10^3^
*C. ulcerans* (cat)	nasal swab	0111326 (99.7 %)	*C.* *ulcerans* (99.5 %)	positive	724
*C. diphtheriae* (PW8)	–	–	–	positive	2×10^3^

Antibiotic susceptibility tests were performed using the broth microdilution method. The isolates showed resistance to clindamycin, but sensitivity to penicillin, cephalosporin, carbapenem, new quinolone and macrolide.

The patient had been raising three cats at home before admission to the hospital. Cats and dogs are recognized as important sources of *C. ulcerans* infection in humans [7–9]. Serum samples, nasal swabs, throat swabs, conjunctival swabs and ear swabs were collected from the cats to determine the source of infection. The serum anti-DT antibody levels of the three cats were analysed by a retrospective toxin neutralization test using Vero cells [5, 6], and resulted in levels of 0.02, 0.056 and 0.08 IU ml^−1^, respectively, indicating that all three cats had histories of infection by toxigenic *C. ulcerans*. Gram-positive bacillus was isolated from the nasal swab of one of the cats. The API Coryne system revealed that the isolate was *C. ulcerans* (API code 0111326 ID 99.7 %). As with the isolates from the patient, the isolates from the cat were determined to be DT-producing *C. ulcerans* by the analysis of the 16S rRNA gene sequence and toxigenicity (Table 1). Analyses using pulsed-field gel electrophoresis (PFGE) and ribotyping were performed as described previously [10–12] to compare the isolates from the patient and the cat. The PFGE patterns and the ribotypes of both isolates matched perfectly (Fig. 2b, c). The nucleotide sequences of the 16S rRNA genes and the DT genes of both isolates were also identical. These data indicated that the patient’s pet cat was the source of infection.

In the same way, the PFGE pattern, the ribotype (Type R1 according to previously published literature [9, 13]), and nucleotide sequences of the 16S rRNA gene and the DT gene of the isolate in this case were also identical to those of the *C. ulcerans* strain 0102 isolated from the first case in Japan in 2001 (Fig. 2b, c).

## Discussion

*C. ulcerans* is closely associated with *Corynebacterium diphtheriae* and was first reported in 1927 by Gilbert and Stewart, who isolated this bacterium from the throat of a patient with a diphtheria-like illness [14]. It has been reported that a DT-non-producing *C. diphtheriae* becomes a DT-producing strain by the infection of bacteriophage [15]. It has been suggested that *C. ulcerans* also possesses the DT gene on a bacteriophage lysogenized in the chromosome [16, 17]. The *C. ulcerans* strains producing DT can cause respiratory illness in humans and animals.

The introduction of the diphtheria toxoid vaccine reduced the number of *C. diphtheriae* infections, and there had been no report of a case of *C. diphtheriae* infection in Japan since 1999. However, reports of human infection with *C. ulcerans* have increased over the last 20 years, and *C. ulcerans* has been recognized as an emerging human pathogen [2]. The first *C. ulcerans* infection in a human in Japan was reported in 2001 [18]. Although cases of *C. ulcerans* infection are increasing [19, 20], there had, to our knowledge, been no fatal case until the present one, the first fatal case of *C. ulcerans* infection in Japan.

The Vero cell cytotoxicity test revealed that the isolate from this fatal case was not more cytotoxic than the strains isolated in the past [21]. The patient had been suffering from depression and had had a history of refusing to see a doctor. It seemed that the patient died because she was not diagnosed at an early stage and therapeutic intervention was delayed. In *C. ulcerans* infection, recovery is likely after antibiotic administration or antitoxin administration during an early stage of infection confined to upper respiratory inflammation, but if diagnosis is delayed, there is a possibility that pseudomembrane will occlude the airway and cause suffocation, as in this case. Early diagnosis and subsequent early treatment are necessary to prevent the progression of this infection.

Diphtheria antitoxin therapy (DAT) is considered effective within three days of the onset of *C. diphtheriae* infection. In Japan, the diphtheria antitoxin is supplied by the national government, but DAT would probably have been ineffective in the present case as more than three days had already passed at the time of diagnosis, and asphyxia caused by pseudomembrane was the direct cause of death. Currently, it is considered that *C. ulcerans* infection can also be prevented by diphtheria toxoid vaccination, but the vaccination history in the present case was not clear. People in Japan are inoculated with diphtheria vaccine in two stages. In the first stage, DTaP-IPV (diphtheria, tetanus, acellular pertussis-inactivated polio vaccine) is given as four times from three to 90 months after birth and an additional vaccination is given 12 to 18 months later. In the second stage, the diphtheria and tetanus vaccine is given at 11 to 12 years of age. The antibody titer of the diphtheria toxin may decrease to below the infection protection level in about ten years after vaccination. Since almost half of the adults in Japan are not expected to have sufficient antibody titer (≧0.1 IU ml^−1^) [22], it is considered preferable to reinoculate them with the toxoid vaccine.

The range of hosts that can serve as a reservoir for *C. ulcerans* is quite broad and includes various animals [2]. Cats and dogs are recognized as important sources of infection in humans [7–9]. In this case, a cat was suspected to be the source of infection, as described above. The patient had been raising three cats at home, and the serum antibody levels of DT were high in all three of the cats and *C. ulcerans* was isolated from a nasal swab of one of them. The PFGE pattern and the ribotype of the isolates from the patient and the cat matched perfectly, which indicates that the source of infection was the cat. Although an adequate epidemiological investigation has not been done, according to an epidemiological survey in the Japanese province of Osaka, 7.5 % of 583 dogs in custody were asymptomatic carriers of *C. ulcerans* [23]. It is necessary to recognize that domestic animals such as cats and dogs could be a source of *C. ulcerans* infection to humans.

The PFGE pattern, the ribotype (Type R1), and nucleotide sequences of the 16S rRNA gene and the DT gene of the *C. ulcerans* isolated from the patient were identical to those of the strain 0102 isolated in Chiba, Japan, in 2001. Our patient lived in Kitakyushu, more than 500 miles away from Chiba. There have been another seven *C. ulcerans* infection cases which were caused by the ribotype R1 as in the present case [9, 13]. These cases were reported from several prefectures in Japan, indicating the possibility that the same molecular type of DT-producing *C. ulcerans* is widespread in Japan.
